# Monthly Summary

**Published:** 1872-12

**Authors:** 


### MONTHLY SUMMARY.

*Training the Nervous System*—Dr. J. M. Winn calls attention, in the pages of an English contemporary, to the truth that the exercise and diet compatible with the best intellectual vigor differs from that adapted to the utmost muscular strength. He remarks:

If we keep in view the difference between the structure of the nervous and muscular tissues, it becomes evident that a somewhat different kind of diet is required for the nutrition of the former from that of the latter. In order to develcpe and support the muscular organization, a large proportion of animal food is requisite; not so, when the object is to supply the waste of the more delicate and subtle tissue of the nervous system. Fatty matter and phosphorous enter largely into the composition of the brain. It has been estimated that the former (brain-wax, or brain-fat, as it has been called) amounts to about one-third, and the latter (phosphorous) to one-twentieth or one-thirtieth of its solid substance. It is now generally believed that exertion of the will and reasoning powers cause an expenditure of the brain-material, and a corresponding exhaustion of the nervous force. However, it must not be forgotten that prolonged bodily exercise exhausts the nervous force, as muscular contractions are commonly brought into action by nervous influence; hence the importance of a student avoiding excessive bodily efforts, which expend that nervous energy so essential for brain-work.

He should take gentle exercise for at least two hours daily in the open air, and his diet should be light, simple and nutritious. Large quantities of animal food and malt liquor conduce rather to somnolency than to mental activity. There is a common saying that "fish feeds the brain," which may depend on the phosphorus it contains; certainly there can be no doubt that some sorts of fish, such as soles, whiting, plaice, etc., are peculiarly suitable to a weak digestion I may incidentally observe that I have found a fish diet especially serviceable for those predisposed to phthisis. A certain amount of rest and sleep, as well as a due supply of healthy blood, are absolutely necessary to maintain the brain in a working condition. Above all, the amount of mental work must be in proportion to the physical strength. I knew an instance of a gentleman who attempted to read ten hours a day, and whilst doing so his competitors got ahead of him; he then wisely reduced his time of work to eight hours. He then recovered his lost ground, and came out a wrangler. There are men of iron constitution who can afford to laugh at these precautions, and who can excel both in mental and bodily exercises. A friend of mine who went in for a public examination surpassed most of his contemporaries, who were his equals in mental capacity, by sheer power of physical endurance.—*Med. & Surg. Reporter.*

---

*A Horrible Story from Paris.*—(*Food Journal*, Oct. 1, 1872).—Three men have been arrested, and are now in charge of the police, for manufacturing and selling *saucissons* (large, or "German" sausages) of the most disgusting composition. Perrin, the chief culprit, lived in a house at Levallois-Perret, a commune just outside the walls of Paris, adjoining Neuilly. A very bad smell frequently invaded the streets, and the inhabitants became alarmed, for the house had a terrible reputation, it having been formerly inhabited by a butcher who was executed for murder and for having sold some of the flesh of his victims for human food. The report was soon current that Perrin, who was a sausage maker, had discovered the remains of buried communists, and was using them in his trade. The storm was gathering over the fellow's head, when a mau named Bonnin appeared before the commissary of police of the place, and declared that he had purchased of Perrin three quintals of sausage, to sell by retail to the masons and fishermen of the neighborhood, and that he had been grossly defrauded, as the meat was in a horrible state of decomposition. The manufacturer and his sausages and meat were immediately seized, and the latter was in such a condition that the persons called in to inspect it were seized with serious vomitings. It turned out on inquiry that these dreadful sausages were composed of the flesh of dogs, cats, and other animals collected by "chiffonniers" in the streets of Paris.—*Med. Times.*

---

*Epithelioma Cured by Creasote.*—Dr. Forne reports, in the *Montpelier Medical*, a case of epithelioma of the upper lip of about four centimeters in diameter, cured by the topical application of creasote. The treatment was commenced by dipping a brush in pure creasote, and having removed any that might drop, the whole surface of the ulcer was lightly but firmly touched with the brush, a certain degree of pressure being used, and the brush retained on the same point a few seconds, so as to insure a thorough application. A piece of lint moistened in a gummy solution of creasote was then laid upon the ulcer. The application caused but slight pain. Three days subsequently it was found that the ulcer had not increased. The application was repeated in the same manner, and five times afterwards, at about the same interval. Cicatrization then commenced, and proceeded rapidly, being favored by a dressing of thin paper moistened with a solution of creasote. Entire recovery took place about six weeks after the treatment was commenced.—*Med. & Surg. Reporter.*

